# Ictal semiology in fronto‐opercular epilepsy: A systematic review

**DOI:** 10.1002/epd2.70257

**Published:** 2026-04-27

**Authors:** Zeynep Gokce‐Samar, Alexandra Montavont, Marion Comajuan, Julitta de Bellescize, Eleni Panagiotakaki, Marietta Papadopoulou, Matthildi‐Athina Papathanasiou‐Terzi, Joseph Toulouse, Alexis Arzimanoglou, Karine Ostrowsky‐Coste

**Affiliations:** ^1^ Department of Pediatric Clinical Epileptology, Sleep Disorders, and Functional Neurology Hospices Civils de Lyon Lyon France; ^2^ Pediatric Epilepsy Unit, Department of Neurology Hospital Sant Joan de Déu Barcelona Spain

**Keywords:** anatomo‐clinical correlation, epilepsy surgery, focal epilepsy, fronto‐opercular seizures, ictal semiology, systematic review

## Abstract

A systematic review of the ictal semiology of fronto‐opercular seizures in focal epilepsy was carried out to assess possible anatomical‐clinical correlations and help guide interpretation of ictal semiology during pre‐surgical evaluation. PubMed and Embase databases were searched using the following keywords: “fronto‐opercular OR frontal opercular OR Frontal operculum OR superior perisylvian OR anterior opercular AND (epilep* or seizure).” The date last searched was November 30th, 2025. Studies were selected if they concerned case series, including at least one fully documented patient and informative case reports with the following criteria: patients who underwent a resection limited to the fronto‐opercular cortex, and/or patients for whom the epileptogenic zone (EZ) was proven by intracranial electroencephalogram (iEEG) with insular cortex exploration, and/or by complementary noninvasive explorations. A total of 21 patients with fronto‐opercular epilepsy from 16 studies were included in the review. iEEG was performed in 15 (71%) patients and resection in 16 (76%) patients. The confidence level in the EZ was very high (Engel IA) for 5 patients, high (Engel I not specified IA or Engel IB) for 12 patients, and moderate for 4 patients. The presence of an aura was found in 67% (*n* = 14/21) of the patients with mainly non‐painful somatosensory sensations. This was more frequent in patients with an EZ in the precentral Rolandic operculum compared to those with an EZ in the prefrontal operculum (*n* = 6 versus *n* = 1, respectively, *p* = 0.016). Ictal signs comprised elementary motor symptoms, speech dysfunction, complex motor behavior, respiratory symptoms, salivation, and laughter, with preserved consciousness. Pure fronto‐opercular epilepsy is rare, consisting mainly of contralateral elementary sensory aura especially for the Rolandic operculum. Common ictal signs are motor orofacial and brachial symptoms with preserved consciousness. Further studies including patients who underwent a successful resection limited to the frontal operculum are needed to confirm these anatomical‐clinical correlations.

**Key points**

- Pure fronto‐opercular seizures are rare. S‐EEG is essential for accurately determining whether seizures originate in the opercular or insular region in which the discharges often spread to the operculum.
- Seizures originating in the fronto‐opercular region are commonly associated with contralateral elementary sensory aura, especially for the Rolandic operculum. The most frequent ictal signs are elementary motor symptoms, speech dysfunction, complex motor behavior, respiratory symptoms, salivation, and laughter, with preserved consciousness.

## 1 INTRODUCTION

**FIGURE 1:**
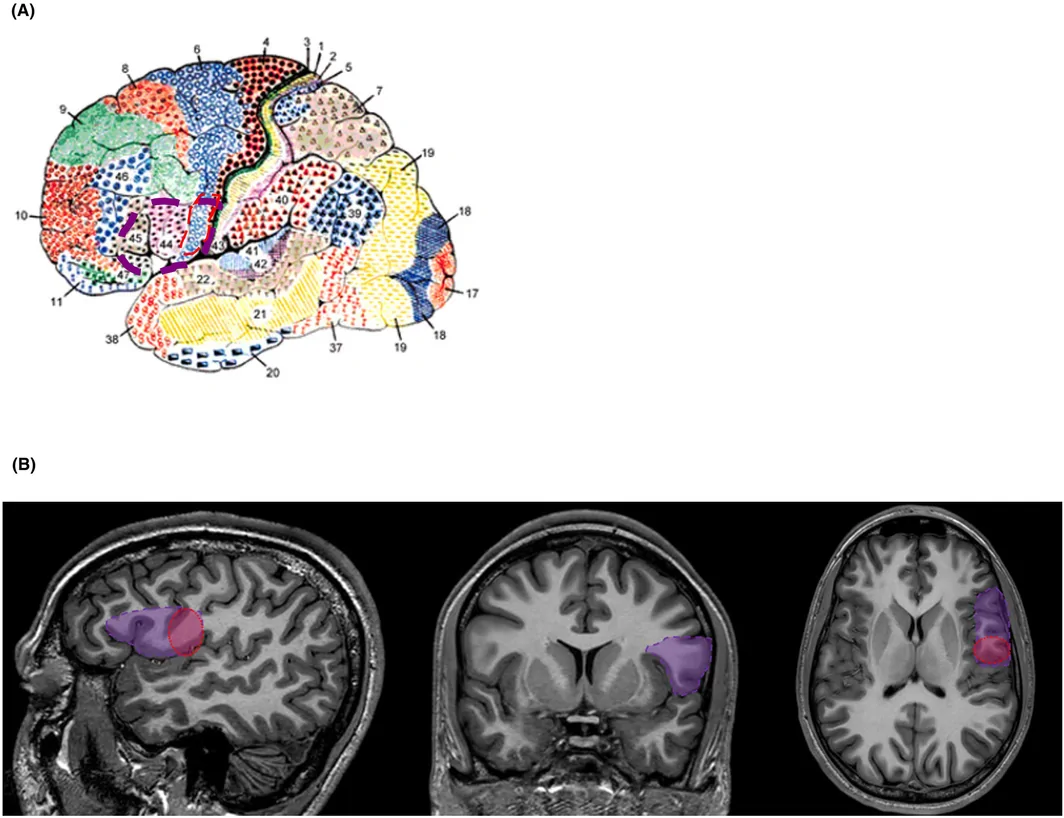
Schematic and magnetic resonance imaging delineation of the fronto‐opercular cortex. Outlined by the thick purple dashed line on a schematic illustration (A) and on MRI (B). The Rolandic operculum is outlined by the red line. (A) Sagittal schematic illustration of the brain with Brodmann's areas (Mesulam)^3^; the fronto‐opercular cortex is delineated by the purple dashed line and the precentral Rolandic operculum by the red dashed line. (B) From left to right: sagittal, coronal, and axial T1‐weighted sequences on brain magnetic resonance imaging; the fronto‐opercular cortex is delineated in purple and the precentral Rolandic operculum in red.

The cerebral operculum covers the insula, and overlaps the frontal, parietal, and temporal lobes adjacent to the Sylvian fissure. The frontal part of the operculum is localized in the middle and posterior parts of the inferior frontal gyrus (F3); it is delimited anteriorly by the posterior and lateral parts of the orbitofrontal cortex, posteriorly by the central sulcus, superiorly by the inferior frontal sulcus, and inferiorly by the Sylvian fissure. In its anterior part, the frontal operculum includes Brodmann's Areas (BA) 45 and 44, which correspond anatomically to the pars triangularis and pars opercularis. In its posterior part, it includes BA 6, corresponding to the Rolandic operculum in the precentral gyrus (Figure 1). The precentral gyrus is somatotopically organized with the face and oral‐lingual‐pharyngeal areas represented at its foot. The frontal operculum therefore straddles the ventrolateral prefrontal cortex (BA 45, 44) and the inferior premotor cortex (lateral BA 6).^1^, ^2^

Opercular seizures are the main seizure subtype of self‐limited epilepsy with centrotemporal spikes (SeLECTs). This well‐defined epileptic syndrome was initially described as benign epilepsy of childhood with centrotemporal spikes by Loiseau and Beaussart in 1958.^4^ Typical manifestations of opercular seizures include oral symptoms, such as sialorrhea and speech difficulties associated with focal clonic movements of the contralateral face.^5^

In non‐idiopathic conditions, the first detailed description of epilepsies of the suprasylvian opercular region was made by Horsley in 1886.^6^ In 1950, Penfield resected a tuber in a 5‐year‐old girl localized in the precentral and postcentral operculum who presented with an ictal semiology of uncontrollable laughter followed by twitching of the right side of the face. Resection led to seizure freedom.^7^ In the early 1960s, Bancaud and Talairach used intracranial electroencephalogram (iEEG) in a large cohort of 210 patients with frontal lobe epilepsy.^2^ The authors identified a small subgroup of 18 patients with seizures originating from the posterior part of the inferior frontal gyrus. At the time, the lack of insular cortex exploration prevented the authors from concluding on a possible origin or participation of the insular cortex in the ictal semiology. The development of stereo‐EEG using transopercular and oblique electrodes has since enabled reaching the insular cortex. A recent study confirmed that while pure opercular epilepsies are less frequent than insulo‐opercular epilepsies, these are more frequent than pure insular epilepsies in which the discharges often spread to the operculum.^8^

We performed a systematic review of fronto‐opercular seizures in non‐idiopathic focal epilepsy, with the aim to summarize the available data in the field and assess possible anatomical and clinical correlations. The present review aimed to help guide interpretation of ictal semiology within the framework of pre‐surgical evaluation.

## 2 METHODS

### 2.1 Search method and eligibility criteria

This systematic review was carried out and reported following the Preferred Reporting Items for Systematic Review and Meta‐Analysis (PRISMA) guidelines. Studies were identified by searching the PubMed and Embase databases using the following keywords: “fronto‐opercular OR frontal opercular OR frontal operculum OR superior perisylvian OR anterior opercular AND (epilep* or seizure),” The date last searched was November 30, 2025. Covidence was used to identify duplicates. Two independent reviewers screened titles, abstracts, and full‐text articles for eligibility criteria. A third reviewer resolved disagreements at the full‐text screening phase.

Case reports and case series published in peer‐reviewed journals in English and French, with an available abstract, were considered for selection. To be selected, studies had to include at least one well‐documented patient or be informative case reports on either patients who underwent a resection limited to the fronto‐opercular cortex and/or patients for whom the epileptogenic zone (EZ) was proven by iEEG with insular cortex exploration and/or by complementary noninvasive explorations (positive magnetic resonance imaging [MRI], source imaging).

### 2.2 Data extraction

For each included study, we extracted the number of reported patients, the results of the explorations (resection, iEEG, MRI), the Engel classification at follow‐up, the EZ (prefrontal operculum or precentral Rolandic operculum), and the ictal signs and symptoms. The risk of bias of the included studies was evaluated using the QUADAS2‐adapted assessment. The level of confidence in the reported EZ was then assessed based on the availability and findings from MRI, iEEG, and post‐operative outcomes. Four levels of confidence were considered: very high, high, moderate, and low. A very high confidence in the reported EZ was defined for patients with Engel class IA after at least 1 year of post‐operative follow‐up. A high confidence concerned patients with either a well delineated focal lesion suspected to represent at least part of the EZ (according to the authors of the publication), or a well delineated EZ according to all available iEEG data (according to the authors of the publication), or an Engel class I (but not specified IA) after at least 1 year of post‐operative follow‐up. A moderate confidence was assigned to patients with MRI signs suspected to be at least part of the EZ. A low confidence was defined for patients with negative MRI or multilobar, multifocal, poorly delineated lesions, or with a poorly delineated EZ according to all available iEEG data (according to the authors of the publication), or an Engel class II–IV after at least 1 year of post‐operative follow‐up in case of complete removal of the EZ.

### 2.3 Statistical analysis

Due to the large heterogeneity in study designs and the low number of patients, quantitative synthesis (meta‐analysis) was not possible. To evaluate significant differences in ictal signs or symptoms according to the EZ (prefrontal operculum or precentral Rolandic operculum), we compared the independent qualitative variables using a Fisher's exact test with a *p*‐value less than 0.05 considered statistically significant.

## 3 RESULTS

### 3.1 Characteristics of included studies

**FIGURE 2:**
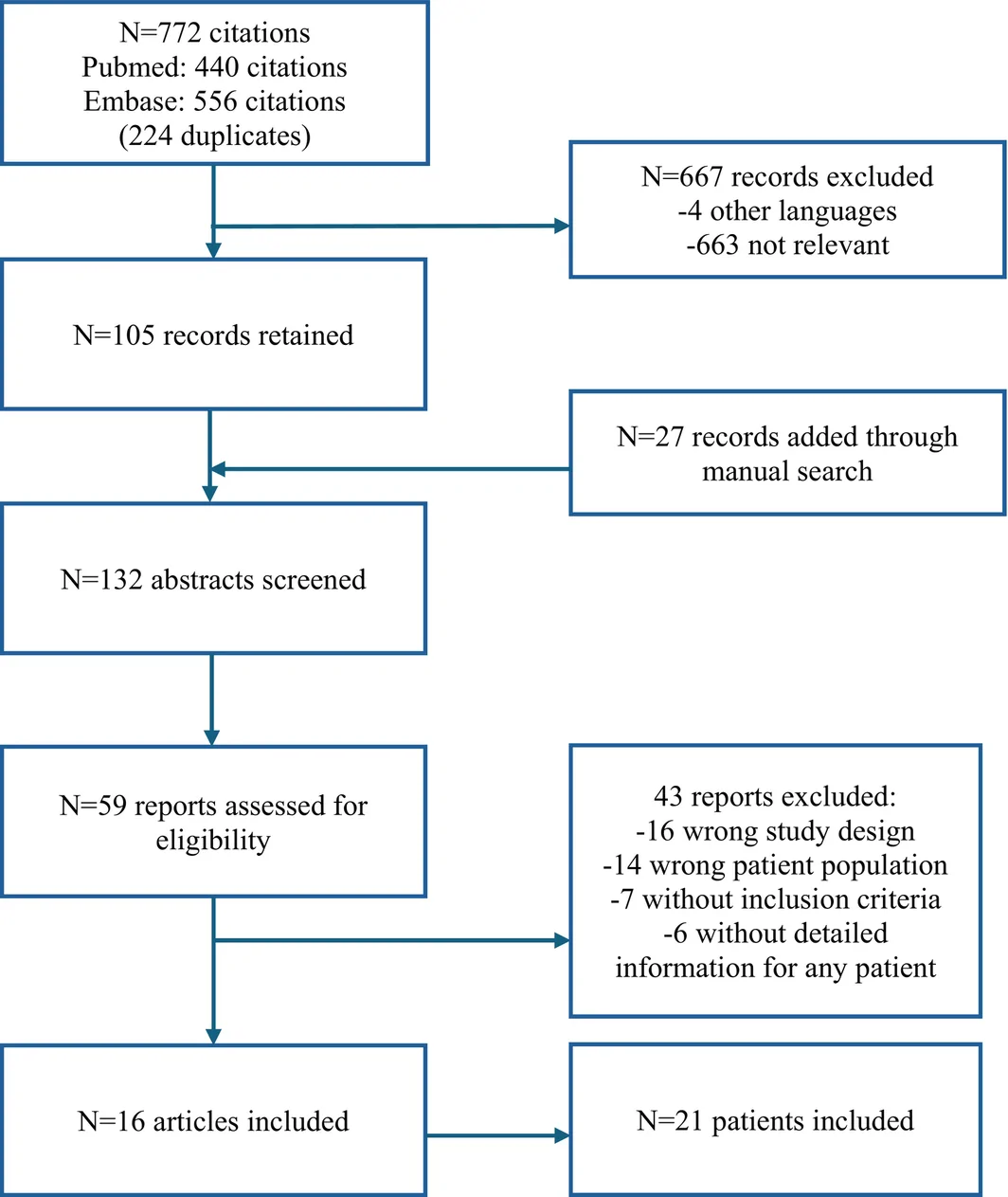
PRISMA flow diagram for the identification, screening, and selection of studies included in the systematic review.

The PRISMA flow diagram (Figure 2) shows that among the 772 citations found (440 citations in PubMed, 556 citations in Embase, 224 duplicates identified through Covidence), 105 abstracts were screened for eligibility, and 27 abstracts were added through manual searching. Among these 132 abstracts, 59 articles were reviewed in full text. Overall, 16 studies representing 21 patients were finally included in the review: nine case series and seven case reports (Table 1).

**TABLE 1:** Characteristics of included studies.

Study (year)	Patients included (*n*)	Adult (A) or pediatric (P) cases (*n*)	Positive MRI (*n*)	iEEG (*n*)	Resection (*n*)	Engel class I ≥1 year (*n*)	Confidence level in the EZ (*n*)
Obana et al. (1992)	2	A (1) P (1)	2	2	2	2	High (2)
Ozlen et al. (1998)	1	A	1	1	1	1	High
Satow et al. (2004)	1	A	1	1	1	1	High
Nobili et al. (2007) and Proserpio et al. (2011)	1	P	1	1	1	1	Very high
Proserpio et al. (2011)	2	A (1) P (1)	2	2	2	2	Very high (1) High (1)
Fluchere et al. (2012)	1	A	0	1	1	1	Very high
Zhou et al. (2016)	2	A (1) P (1)	2	0	1	1	High (1) Moderate (1)
Wang X et al. (2019)	1	A	0	1	1	1	High
Hu et al. (2020)	1	P	0	1	0	NC	High^a^
Wang H et al. (2020)	1	A	NA	1	1	NA	High
Rini et al. (2020)	1	A	0	0	0	NC	Moderate
Chassoux et al. (2022)	3	A (1) P (2)	0	3	3	3	Very high (1) High (2)
Oliveira et al. (2024)	1	A	1	0	1	1	Very high
Chartrain et al. (2024)	1	P	1	0	1	1	High
Paredes‐Aragon et al. (2024)	1	A	0	1	0	NC	Moderate
Di Giacomo et al. (2024)	1	A	1	0	0	NC	Moderate
Total from 16 studies	21	A (13) P (8)	12	15	16	15	Very high (5) High (12) Moderate (4)

*Note*: A total of 21 patients (13 adults and 8 children) from 16 studies were included. Confidence in the EZ was considered very high if patients were Engel IA after at least 1 year of post‐operative follow‐up, high if patients were Engel I (but not specified IA) or Engel IB, and moderate if patients had MRI signs suspected to be at least part of the EZ.

Abbreviations: EZ, epileptogenic zone; iEEG, intracranial EEG; MRI, magnetic resonance imaging; NA, not available; NC, not concerned.

^a^ Confidence level was considered high despite the lack of resection because the patient was seizure‐free 7 months after radio‐frequency‐thermo‐coagulation.

Among all studies, five used a region as an inclusion criterion and included between Patients 1 and 3 (Nobili et al.,^9^ one patient who was later included in the publication of Proserpio et al.^10^; Wang X et al.,^8^ one patient; Wang H et al.,^11^ one patient; Chassoux et al.,^12^ three patients; and Obana et al.,^13^ two patients). The risk of bias of these studies was low thanks to the systematic and successive recruitment according to the brain region.

Four studies used a symptom as an inclusion criterion and included between Patients 1 and 2 (Proserpio et al.,^10^ two patients, excluding the one previously published in Nobili et al.^9^; Fluchere et al.,^14^ one patient; Zhou et al.,^15^ two patients; and Hu et al.,^16^ one patient). The risk of bias of these studies was moderate due to the possibility of subjective assessment of symptoms.

The remaining seven studies were case reports, including one patient each (Ozlen et al.^17^; Satow et al.^18^; Rini et al.^19^; Oliveira et al.^20^; Chartrain et al.^21^; Paredes‐Aragon et al.^22^; and Di Giacomo et al.^23^).

### 3.2 Characteristics of included patients

Overall, all included patients had an MRI, 15 (71%) underwent iEEG, and 16 (76%) had a resection. Of note, one patient was seizure‐free after radio‐frequency‐thermo‐coagulation (RFTC) with a follow‐up of 7 months (Hu et al.^16^).

The confidence in the EZ was very high (Engel IA) for five patients (Nobili et al.^9^; Proserpio et al.^10^; Fluchere et al.^14^; Chassoux et al.^12^; and Oliveira et al.^20^), high (Engel I but not specified IA or Engel IB) for 12 patients (Obana et al.,^13^ Ozlen et al.,^17^ Satow et al.,^18^ Proserpio et al.,^10^ Zhou et al.,^15^ Wang X et al.,^8^ Wang H et al.,^11^ Chassoux et al.,^12^ Chartrain et al.^21^), and moderate for four patients (Zhou et al.^15^; Rini et al.^19^; Di Giacomo et al.^23^; and Paredes‐Aragon et al.^22^). The patients reported by Zhou et al., Rini et al., and Di Giacomo et al. did not have pre‐surgical or surgical investigations but had focalized abnormalities in the frontal‐opercular regions on brain imaging. The patient reported by Paredes Aragon et al. had iEEG but without RFTC nor resection. Overall, the summary of evidence was considered of high reliability.

### 3.3 Anatomical and clinical correlations

The semiological analysis of all included patients showed the presence of an aura in 67% (*n* = 14/21) of the patients with non‐painful somatosensory sensations mostly of the face or the contralateral arm in 7 (33%) patients, viscero‐sensory sensations, including oral‐lingual‐pharyngeal and epigastric sensations in five (24%) patients, and palpitations in two (10%) patients. Auras were not composed of mixed symptoms but were mostly described as a unique type of sensation.

Ictal signs observed at onset or during early propagation of the seizure comprised elementary motor symptoms, speech dysfunction, complex motor behavior with hyperkinetic automatisms, respiratory symptoms, salivation, vegetative signs, laughter, and facial emotion. The elementary motor symptoms were the most frequent, occurred early in the seizure, and concerned mainly the orofacial regions (43%, *n* = 9) and/or the arms (62%, *n* = 13). In the arms, these elementary motor symptoms were mainly contralateral tonic and/or clonic seizures or dystonic posture. Bilateral asymmetric tonic posture, which could occur at onset or during propagation, was less frequently observed (24%). Speech dysfunction was reported as anarthria in both hemispheres (29%, *n* = 6). Complex motor behavior with hyperkinetic automatisms occurred in 24% (*n* = 5) of the patients. Respiratory symptoms such as dyspnea and hyperventilation were reported in 29% (*n* = 6) of the patients and salivation in 19% (*n* = 4). Vegetative signs such as tachycardia, bradycardia, and rubefaction were described in 25% (*n* = 5) of cases. Finally, laughter occurred in 14% (*n* = 3) of the patients and facial emotion, described early in the seizure as facial distress, in 10% (*n* = 2). In the majority of patients (62%, *n* = 13), consciousness was preserved. During seizure propagation, impaired consciousness was observed in three patients (35%), vocalization (grunts) in two patients (12%), and negative motor signs in two patients (12%; one patient with hand weakness and another with contralateral arm flaccid paralysis). Focal to bilateral tonic–clonic seizures were unfrequent (19%, *n* = 4).

An analysis of ictal semiology was then carried out according to the EZ by separating the frontal operculum into two groups: One group of 12 patients with an EZ located in the prefrontal operculum (Areas 44 and 45, the pars triangularis and opercularis, respectively) and another group of 9 patients whose EZ concerned the precentral Rolandic operculum (the lower part of Area 6). The only significant difference between these two groups was the more frequent presence of somatosensory aura in the Rolandic operculum group compared with the prefrontal operculum group (*n* = 6 versus *n* = 1, respectively, *p* = 0.016). No other significant difference in semiological features of the seizures was identified (Table 2).

**TABLE 2:** Anatomical and clinical correlations.

Ictal sign or symptom	Reported in frontal operculum, *N* = 21, *n* (%)	Reported more at onset or during propagation	Overall level of agreement that the sign/symptom is suggestive of fronto‐opercular epilepsy	Reported in prefrontal operculum, *N* = 12, *n*	Reported in precentral Rolandic operculum, *N* = 9, *n*	*p*‐value
No aura	7 (33)		Low	7	0	**0.007**
Aura	14 (67)	Onset	High	5	9	**0.007**
Somatosensory (arm and/or face)	7 (33)	Onset	High	1	6	**0.016**
Viscero‐sensory	5 (24)	Onset	High	3	2	1
Oral‐lingual‐pharyngeal	3 (14)	Onset	High	2	1	1
Epigastric	2 (10)	Onset	Low	1	1	1
Autonomic aura (palpitation)	2 (10)	Onset	Low	1	1	1
Ictal semiology	21 (100)			12	9	1
Elementary motor: orofacial (tonic and/or clonic, grimace)	9 (43)	Onset	High	4	5	0.396
Elementary motor: other	13 (62)	Onset	Moderate	6	7	0.367
Brachial contralateral (tonic and/or clonic or dystonic posture)	8 (38)	Onset	Moderate	4	4	0.673
Bilateral asymmetric tonic posture	5 (24)	Both	High	2	3	0.611
Negative motor	2 (10)	Late	High	0	2	0.171
Complex motor behavior (hyperkinetic automatisms)	5 (24)	Both	Moderate	3	2	1
Salivation	4 (19)	Both	High	2	2	1
Choking, dyspnea, apnea, throat constriction	5 (24)	Onset	High	2	3	0.611
Hyperventilation	1 (5)	Onset	Low	1	0	1
Tachycardia	2 (10)	Both	Low	1	1	1
Bradycardia	1 (5)	Late	Low	0	1	0.429
Rubefaction/flushing	2 (10)	Both	Low	1	1	1
Laughter (mirthless)	3 (14)	Both	Low	2	1	1
Speech dysfunction	6 (29)	Both	Moderate	2	4	0.331
Vocalization (grunts)	2 (10)	Late	Low	2	0	0.486
Facial distress	2 (10)	Onset	Low	2	0	0.486
Preserved consciousness	13 (62)	Both	High	8	5	0.673
Impaired consciousness	8 (38)	Late	Low	4	4	0.673
Focal to bilateral tonic–clonic seizure	4 (19)	Late	Low	3	1	0.603

*Note*: List of ictal symptoms and signs according to the precise localization in the frontal operculum. The most frequent ictal signs were the presence of an aura (67%), brachial and orofacial elementary motor signs (62% and 43%, respectively), preserved consciousness (62%), speech dysfunction (29%), choking or dyspnea (24%), and hyperkinetic automatisms (24%). A significant difference in the presence of an aura, especially the somatosensory aura, was noted with a higher frequency in the precentral Rolandic operculum compared with the prefrontal operculum.

## 4 DISCUSSION

The present systematic review included 21 patients with fronto‐opercular epilepsy from 16 studies (nine case series and seven case reports). To be considered, studies had to include at least one well‐documented patient or be informative case reports according to the following criteria: patients had to be treated by a resection limited to the fronto‐opercular cortex (Engel I), and/or have an EZ within the frontal‐opercular cortex either proven by iEEG with insular cortex exploration, and/or by complementary noninvasive explorations. The main limitation of the present systematic review was thus the very low number of patients meeting these inclusion criteria and the lack of detailed descriptions of seizure semiology in most of the surgical series. Nonetheless, more than 70% of the included patients underwent iEEG and/or resection, leading to an overall high reliability of the summarized evidence, thereby increasing the relevance of the anatomical and clinical correlations.

The present review confirms that fronto‐opercular seizure often presents with somatosensory aura affecting the contralateral face or arm or a viscero‐sensory aura concerning the oropharyngeal organs. The ictal sign sequence also comprises elementary motor signs, such as contralateral face or arm tonic or clonic movements (or a bilateral asymmetric tonic posture to a lesser degree) that are more present at onset and associated with salivation, respiratory disorders, and speech dysfunction. Consciousness is often preserved. Of note, preserved consciousness is frequently observed in idiopathic SeLECTs, where seizures are thought to arise from the opercular cortex.^24^

When comparing the semiology of patients according to a more precisely delineated anatomical location (i.e., between epilepsy originating in the prefrontal operculum and epilepsy originating in the precentral Rolandic operculum), we found a significant difference only for somatosensory aura, which was more common in Rolandic operculum epilepsy. This is probably due to the proximity and interconnection of this region with the primary somatosensory areas. Although no other significant difference was found between the two groups, it should be noted that signs, such as hyperventilation, vocalization, and facial distress were never reported in patients with Rolandic operculum epilepsy.

Regarding the elementary motor signs, focal clonic seizures of the face have a highly localizing value, always involving the contralateral central (Rolandic) operculum, which is linked to the frontal operculum in terms of cytoarchitectonics.^25^, ^26^ The Rolandic operculum produces perioral muscle clonic or tonic movements or contractions. Wang et al. showed a strong link between facial symptoms and the Rolandic operculum but not with other cortical areas.^27^ While elementary motor signs were the most frequent objective motor signs observed herein, no significant difference in these signs was observed between the prefrontal operculum group and the precentral Rolandic operculum group. Elementary motor signs occur early in the seizure and affect the upper limbs and/or the orofacial region contralaterally to the EZ with tonic, clonic, or dystonic movements. This symptomatology has been linked to the spread of the ictal discharge outside of the insulo‐opercular complex^28^ and with seizures arising from the most posterior and caudal part of the frontal lobe.^29^

The findings of the present study are concordant with the early description made by Bancaud and Talairach^2^ of a subgroup of 18 patients who presented with seizures arising from the posterior part of the inferior frontal gyrus. These 18 patients were not included herein due to the lack of insular cortex exploration. However, the authors also described contralateral elementary motor orofacial and brachial symptoms and occasional postural motor manifestations with brachial predominance and speech dysfunction. Salivation and respiratory symptoms, including apnea, were also frequent, as were vegetative signs such as tachycardia and facial rubefaction. Of note, the authors also reported mydriasis and pallor, which were not found in the present review.

Differentiating between insular and opercular involvement during insulo‐opercular epilepsy^10^, ^11^, ^30^, ^31^ is difficult, as both may be involved simultaneously in seizure onset. To our knowledge, only two series^28^, ^32^ specifically aimed at differentiating pure insular semiology from opercular semiology. Peltola et al.^32^ and Singh et al.^28^ used iEEG with both orthogonal and oblique electrode implantations, and described 11 and 9 patients with an EZ strictly limited to the insula, respectively. In the present systematic review, half of the patients with an aura reported non‐painful paresthesia contralateral to the EZ at the beginning of their seizures. The latter was limited to the face or upper limb and was more frequent in the precentral Rolandic operculum group. Interestingly, in the series by Peltola et al., eight (72%) patients had non‐painful paresthesia, four patients with an EZ in the anterior insula and four in the posterior insula. Similarly, three (33%) patients from the series by Singh et al. had face and hand paresthesia. These findings appear to suggest that the occurrence of non‐painful paresthesia does not discriminate between insula and frontal operculum involvement. However, paresthesia in these series is described as more extensive, variable, and/or affecting multiple territories or limbs while in the present review, a Rolandic opercular involvement was found to lead to paresthesia limited to the face or the upper limb. Another aura characteristic of pure opercular involvement identified herein was the sensation of laryngeal constriction. This symptom was found in only 1/11 (9%) patient from the study by Peltola et al.^32^ and in none of the patients reported by Singh et al.^28^

Regarding other ictal signs, hypersalivation and respiratory disorders were found in nearly half of the patients herein, but never described in the pure insular patients of Peltola et al.^32^ and Singh et al.^28^ It only occurred in five (45%) patients of Peltola et al.,^32^ in whom the ictal discharge spread outside the insular cortex, remaining “peri‐sylvian,” and thus presumed to be an insulo‐opercular discharge. This suggests that whether the onset of the epileptic discharge is insular (with secondary opercular spread) or purely opercular, hypersalivation and respiratory disorders occur at the time of the opercular involvement.

Speech arrest (anarthria or dysarthria) can be the first sign of seizures starting from the frontal operculum^2^; language disorders were indeed found in nearly a third of the patients herein. Alone, anarthria is not enough to designate a hemispheric lateralization of the discharge. It essentially means a disturbance of the phonatory musculature. In the dominant hemisphere, seizures can produce the same symptoms as in the non‐dominant hemisphere, in addition to producing dysphasia, which may persist into the postictal period. These symptoms may precede focal clonic or tonic movements affecting the face and arm in a Jacksonian way.^2^ The occurrence of language disorders was also reported by Peltola et al., who described symptoms combining motor aphasia, dysprosody, or dysarthria occurring early in seizure semiology but not at initial onset, once again suggesting an opercular spread of the discharge.

Regarding the timing of the seizures, very little information was provided in the studies included in the present review. Only the study by Nobili et al.^9^ clearly specified that seizures occurred at night for one patient who presented with hyperkinetic seizures. Complex motor behaviors with a hyperkinetic aspect, found in a fourth of the patients herein, are also reported in frontal, parietal, temporal, and insular epilepsies and are therefore not specific to opercular involvement.^33^, ^34^, ^35^, ^36^ Finally, two patients in our series were described as presenting with laughter or a sudden outburst of mirthless laughter as has been rarely described in fronto‐opercular epilepsies.^37^

## 5 CONCLUSION

Pure non‐idiopathic fronto‐opercular epilepsy is rare as reflected by the 21 patients included in this systematic review. Most of them benefited from iEEG and resection, increasing the confidence in the EZ and enabling us to make relevant anatomical and clinical correlations. Fronto‐opercular seizures semiology mostly consists of a somatosensory aura of the contralateral face and/or arm, especially when seizures arise from the Rolandic part of the frontal operculum. Viscero‐sensory aura involving oral‐lingual‐pharyngeal organs is also reported. The most frequent objective ictal signs are contralateral elementary motor orofacial and/or brachial symptoms, speech dysfunction, salivation, respiratory disorders, with mostly preserved consciousness. Less frequently, hyperkinetic motor behaviors can also be found, as well as laughter.

## FUNDING INFORMATION

The authors have nothing to report.

## CONFLICT OF INTEREST STATEMENT

All authors had no conflicts of interest to disclose.

## Supporting information


Appendix S1.

## Data Availability

The data that support the findings of this study are available from the corresponding author upon reasonable request.
