# Supplementary material for: Ictal semiology in fronto‐opercular epilepsy: A systematic review
Source: Epileptic Disord. 2026 Apr 27;28(4):1047–57. doi: 10.1002/epd2.70257 (PMC13499277; doi:10.1002/epd2.70257)
Supplement: Supplementary file 1 [file EPD2-28-1047-s001.docx]

**TEST YOURSELF**

**Answers**

**1. B, C, D**

**2. A, B, C, D**

**3. A**
